# An ensemble *n*-sub-epidemic modeling framework for short-term forecasting epidemic trajectories: Application to the COVID-19 pandemic in the USA

**DOI:** 10.1371/journal.pcbi.1010602

**Published:** 2022-10-06

**Authors:** Gerardo Chowell, Sushma Dahal, Amna Tariq, Kimberlyn Roosa, James M. Hyman, Ruiyan Luo

**Affiliations:** 1 Department of Population Health Sciences, School of Public Health, Georgia State University, Atlanta, Georgia, United States of America; 2 Division of International Epidemiology and Population Studies, Fogarty International Center, National Institutes of Health, Bethesda, Maryland, United States of America; 3 National Institute for Mathematical and Biological Synthesis (NIMBioS), University of Tennessee, Knoxville, Tennessee, United States of America; 4 Department of Mathematics, Center for Computational Science, Tulane University, New Orleans, Louisiana, United States of America; University of Notre Dame, UNITED STATES

## Abstract

We analyze an ensemble of *n*-sub-epidemic modeling for forecasting the trajectory of epidemics and pandemics. These ensemble modeling approaches, and models that integrate sub-epidemics to capture complex temporal dynamics, have demonstrated powerful forecasting capability. This modeling framework can characterize complex epidemic patterns, including plateaus, epidemic resurgences, and epidemic waves characterized by multiple peaks of different sizes. We systematically assess their calibration and short-term forecasting performance in short-term forecasts for the COVID-19 pandemic in the USA from late April 2020 to late February 2022. We compare their performance with two commonly used statistical ARIMA models. The best fit sub-epidemic model and three ensemble models constructed using the top-ranking sub-epidemic models consistently outperformed the ARIMA models in terms of the weighted interval score (WIS) and the coverage of the 95% prediction interval across the 10-, 20-, and 30-day short-term forecasts. In our 30-day forecasts, the average WIS ranged from 377.6 to 421.3 for the sub-epidemic models, whereas it ranged from 439.29 to 767.05 for the ARIMA models. Across 98 short-term forecasts, the ensemble model incorporating the top four ranking sub-epidemic models (Ensemble(4)) outperformed the (log) ARIMA model 66.3% of the time, and the ARIMA model, 69.4% of the time in 30-day ahead forecasts in terms of the WIS. Ensemble(4) consistently yielded the best performance in terms of the metrics that account for the uncertainty of the predictions. This framework can be readily applied to investigate the spread of epidemics and pandemics beyond COVID-19, as well as other dynamic growth processes found in nature and society that would benefit from short-term predictions.

## Author summary

The COVID-19 pandemic has highlighted the urgent need to develop reliable tools to forecast the trajectory of epidemics and pandemics in near real-time. We describe and apply an ensemble *n*-sub-epidemic modeling framework for forecasting the trajectory of epidemics and pandemics. We systematically assess its calibration and short-term forecasting performance in weekly 10–30 days ahead forecasts for the COVID-19 pandemic in the USA from late April 2020 to late February 2022 and compare its performance with two different statistical ARIMA models. This framework demonstrated reliable forecasting performance and substantially outcompeted the ARIMA models. The forecasting performance was consistently best for the ensemble sub-epidemic models incorporating a higher number of top-ranking sub-epidemic models. The ensemble model incorporating the top four ranking sub-epidemic models consistently yielded the best performance, particularly in terms of the coverage rate of the 95% prediction interval and the weighted interval score. This framework can be applied to forecast other growth processes found in nature and society, including the spread of information through social media.

## Introduction

The coronavirus disease 2019 (COVID-19) pandemic has amplified the critical need for reliable tools to forecast the trajectory of epidemics and pandemics in near real-time. During the early stages of the COVID-19 pandemic, multiple modeling teams embarked on the challenging task of producing short-term forecasts of the course of the COVID-19 pandemic in terms of the trajectory for the number of new cases, hospitalizations, or deaths (e.g., [1–10]). Soon after the epidemic started, our research team published short-term forecasts of the pandemic during the early outbreaks of the novel coronavirus in China [4] and subsequently focused on producing weekly forecasts for the USA [11]. In a related effort, the US COVID-19 Forecasting Hub brought together multiple research teams to synthesize weekly short-term forecasts of the COVID-19 pandemic in the USA [12]. It is important to evaluate rigorously the forecasting performance of these different pandemic forecasting efforts and document the lessons learned to continue advancing our understanding of epidemic forecasting.

Ensemble modeling approaches and models that integrate sub-epidemics to capture complex temporal dynamics have demonstrated powerful forecasting capability (e.g., [13–17]). In prior work, we developed a sub-epidemic modeling framework to characterize and improve forecasting accuracy during complex epidemic waves [13]. This mathematical framework characterizes epidemic curves by aggregating multiple asynchronous sub-epidemics and outperforms simpler growth models in providing short-term forecasts of various infectious disease outbreaks [13,18]. It is possible to model sub-epidemics associated with transmission chains that are asynchronously triggered and progress somewhat independently from the other sub-epidemics. This framework supports a family of sub-epidemic models that yield similar fits to the calibration data, but their corresponding forecasts could produce diverging trajectories.

Ensemble modeling aims to boost forecasting performance by systematically integrating the predictive accuracy tied to individual models [16,19–21]. Past work indicates that multimodel ensemble approaches are powerful forecasting tools that frequently outperform individual models in epidemic forecasts [14,15,22–27]. We extend prior sub-epidemic modeling work and propose an ensemble sub-epidemic modeling framework for forecasting the trajectory of epidemics and pandemics. In this model, the sub-epidemics can start at different time points and may differ in growth rates, scaling of growth, and sub-epidemic size parameters. The individual sub-epidemics are frequently unobserved and shaped by multiple heterogeneities such as asynchronous focal transmission occurring in different spatial areas, the transmission burden gradually shifting from high-risk to lower-risk groups [28], varying intensity of public health interventions over time, and the emergence of new variants of the pathogen, to name a few. Hence, this ensemble modeling framework can characterize more diverse epidemic patterns, including plateaus, epidemic resurgences, and epidemic waves characterized by multiple peaks of different sizes, which were impossible to capture in earlier sub-epidemic frameworks [13].

We systematically assess the calibration and short-term forecasting performance in weekly 10–30 day forecasts in the context of the COVID-19 pandemic in the USA from late April 2020 to late February 2022, including the Omicron-dominated wave. We then compare the performance of the ensemble modeling framework with a set of Autoregressive Integrated Moving Average (ARIMA) models, following the EPIFORGE 2020 guidelines to report epidemic forecasts [29]. Our extended ensemble modeling framework substantially outperforms individual top-ranking sub-epidemic models and the ARIMA models based on standard performance metrics that account for the uncertainty of the predictions.

## Results

### Quality of the sub-epidemic model fits

**Fig 1:**
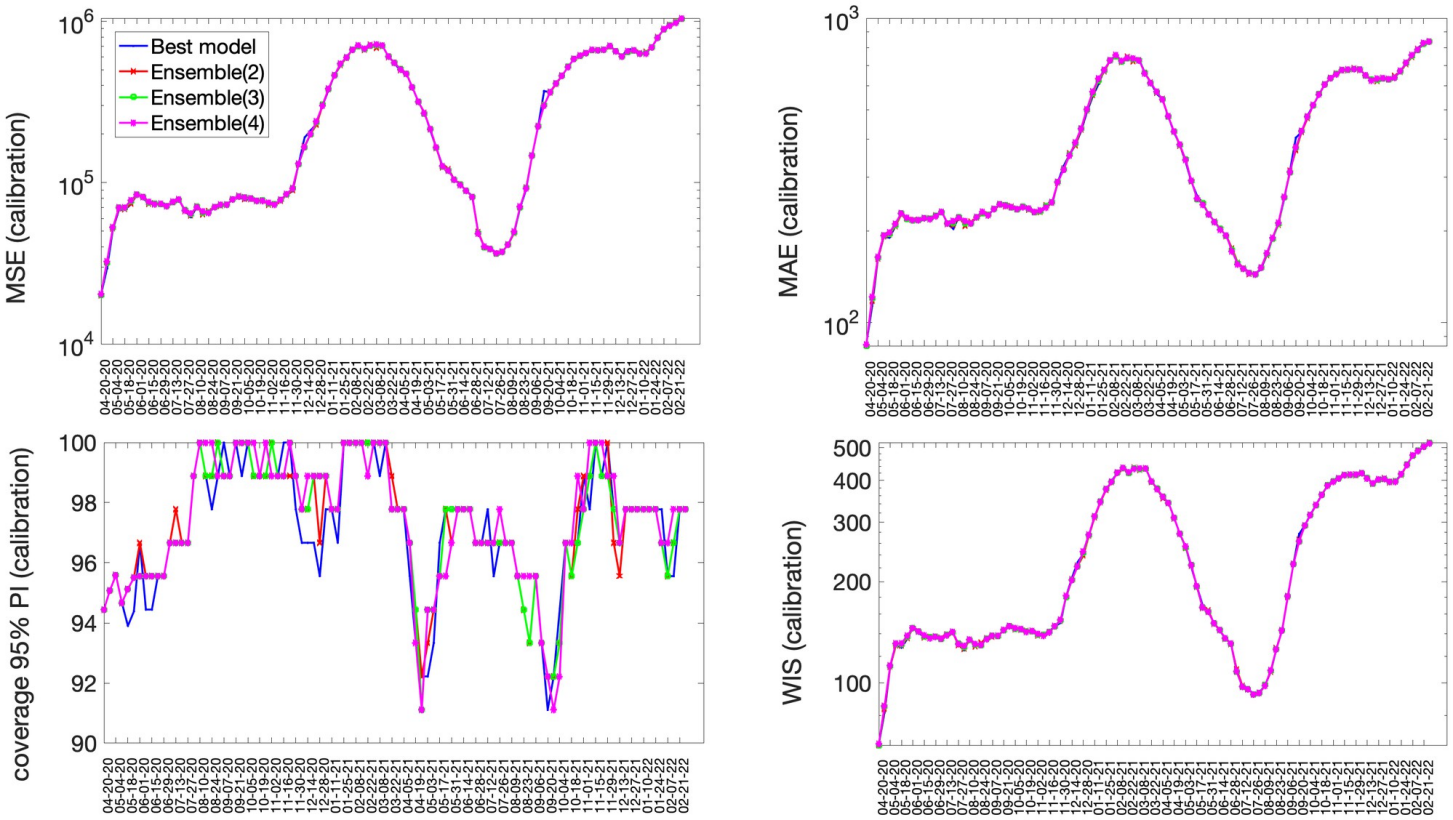
Performance metrics quantifying the quality of the sub-epidemic model fits to 98 sequential weekly calibration periods of the daily time series of COVID-19 deaths in the USA from 20-April-2020 through 22-February 2022. The best fit sub-epidemic model and three ensemble models constructed using the top-ranking sub-epidemic models (Ensemble(2), Ensemble(3), Ensemble(4)) yielded similar quality fits.

The best fit sub-epidemic model and three ensemble models constructed using the top-ranking sub-epidemic models (Ensemble(2), Ensemble(3), Ensemble(4)) yielded similar quality fits to 98 sequential weekly calibration periods from 20-April-2020 to 28-February-2022 (Fig 1 and Table 1). For instance, the average WIS was ~247 with slight variation across models (Table 1). The coverage rate of the 95% PIs averaged 97% and ranged from 91% to 100% during the study period. Moreover, all performance metrics displayed similar temporal trends (Fig 1).

**Table 1:** Mean performance metrics quantifying model fit quality across 98 sequential weekly calibration periods of the daily time series of COVID-19 deaths in the USA from 20-April-2020 through 22-February 2022.

Model	Mean absolute error (MSE)	Mean squared error (MAE)	Percentage coverage of the 95% prediction interval	Weighted Interval Score (WIS)
Best fit sub-epidemic model	309260.00	394.74	97.06	247.28
Ensemble(2) model	308300.00	394.91	97.30	246.93
Ensemble(3) model	308620.00	395.24	97.46	247.09
Ensemble(4) model	309160.00	396.17	97.46	247.33

*The Ensemble(*i*) model incorporates the top *i* ranked sub-epidemic models in the ensemble as described in the text.

**Fig 2:**
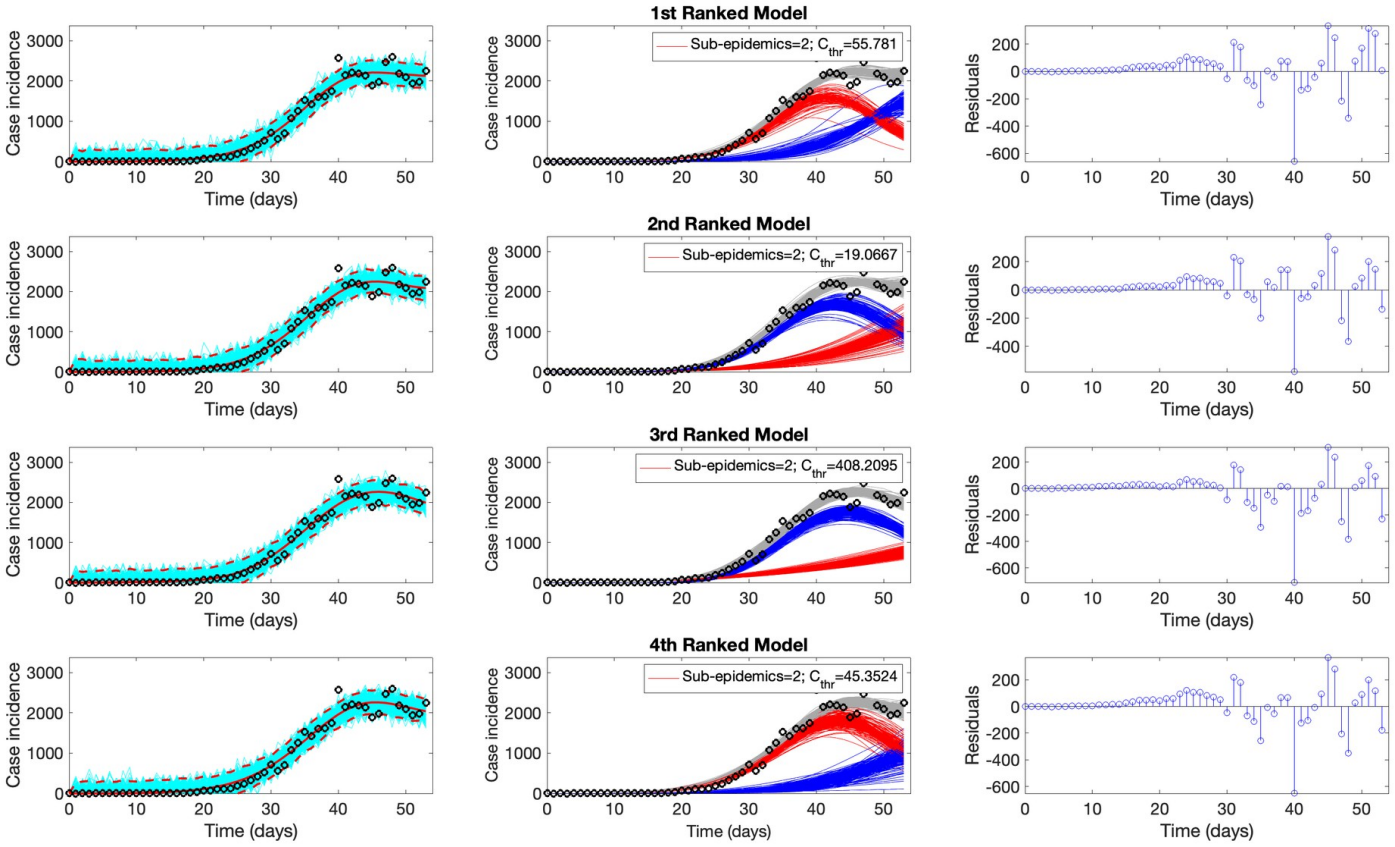
Representative fits of the top-ranking sub-epidemic models to the daily curve of COVID-19 deaths in the USA from 27-Feb-2020 to 20-April-2020. The sub-epidemic models capture well the entire epidemic curve, including the latter plateau dynamics, by considering models with two sub-epidemics. The best model fit (solid red line) and 95% prediction interval (dashed red lines) are shown in the left panels. The cyan curves correspond to the associated uncertainty from individual bootstrapped curves. The sub-epidemic profiles are shown in the center panels, where the red and blue curves represent the two sub-epidemics, and the grey curves are the estimated epidemic trajectories. For each model fit, the residuals are also shown (right panels). Black circles correspond to the data points.

**Fig 3:**
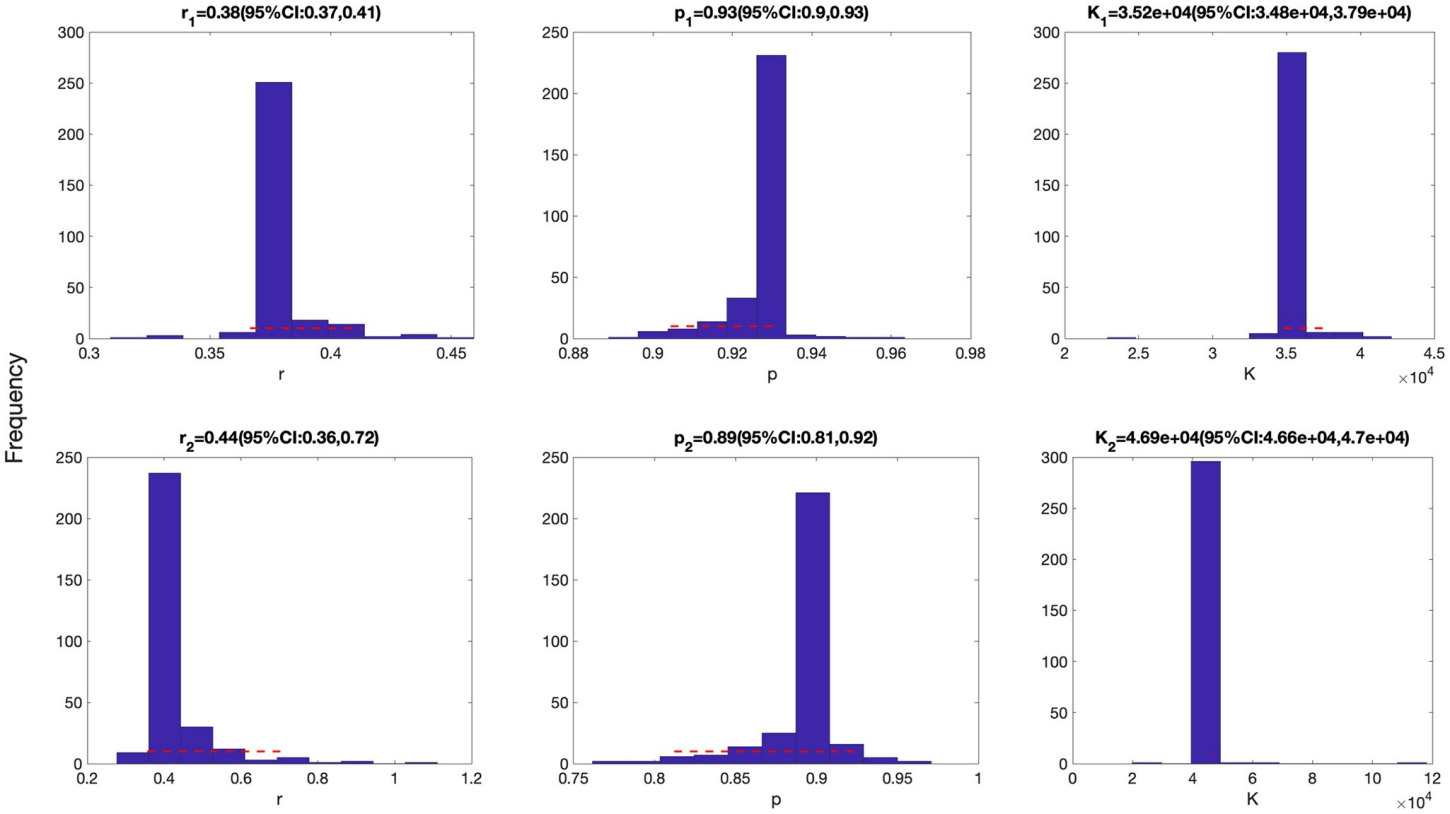
Parameter estimates for the first (top panel) and the second sub-epidemics (bottom panels) were derived for the top-ranking sub-epidemic model after fitting the sub-epidemic modeling framework to the daily curve of COVID-19 deaths in the USA from 27-Feb-2020 to 20-April-2020 (see also Fig 1). Parameter estimates for both sub-epidemics are well identified, as indicated by their relatively narrow bootstrap confidence intervals.

Representative fits of the top-ranking sub-epidemic models to the daily curve of COVID-19 deaths in the USA from 27-Feb-2020 to 20-April-2020 are shown in Fig 2. Although these sub-epidemic models fit the data well, each results from the aggregation of two sub-epidemics characterized by different growth rates, scaling of growth, and outbreak sizes, as shown in Fig 3.

### Short-term forecasting performance

**Fig 4:**
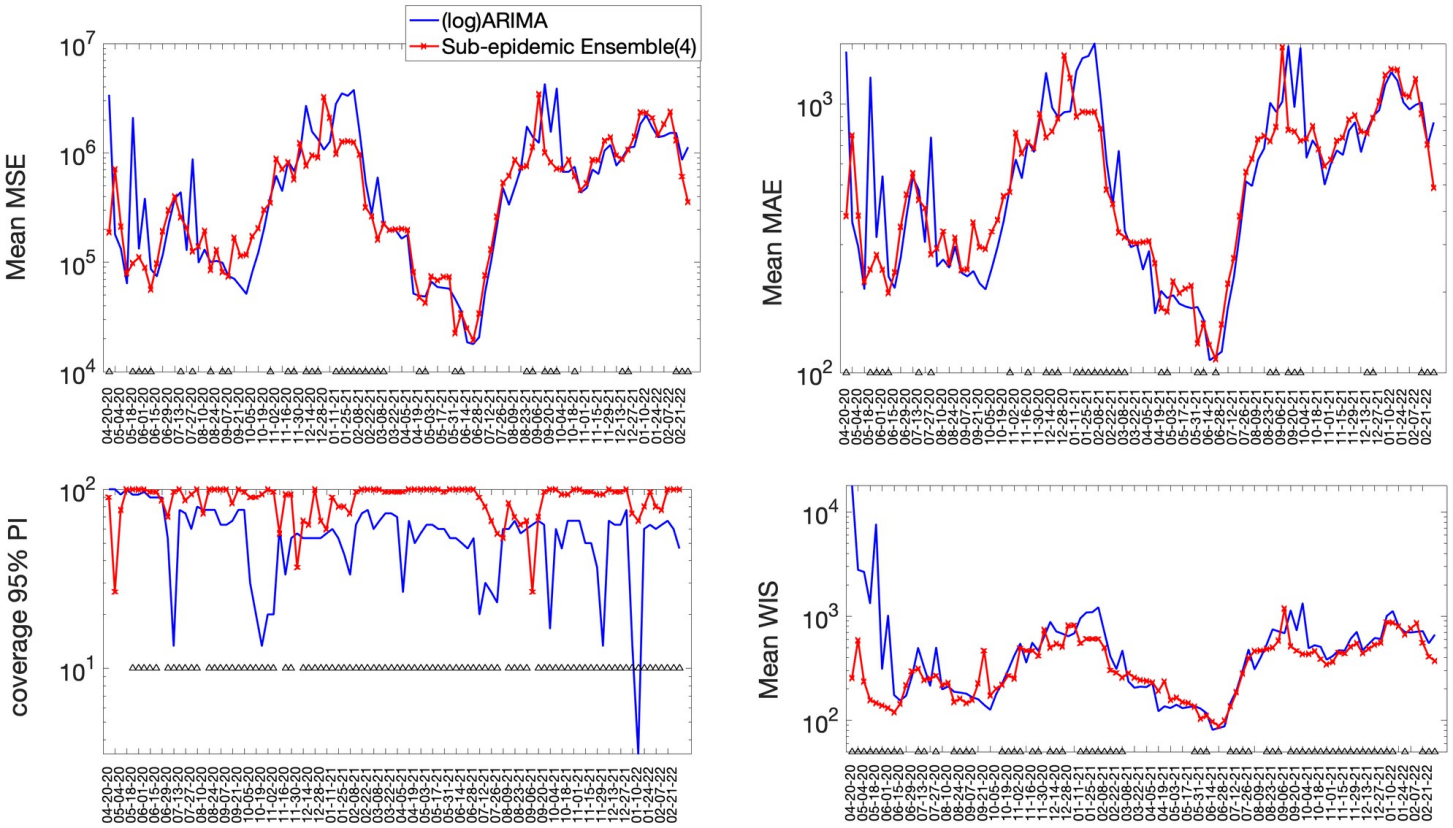
Forecasting performance metrics for the (log) ARIMA and Ensemble(4) models across 98 30-day forecasts. The symbol (^) indicates weekly forecasts where the Ensemble(4) model outperformed the (log) ARIMA model. For example, the Ensemble(4) outperformed the (log) ARIMA model 66.3% of the time in terms of the WIS and 89.8% of the time in terms of the coverage rate of the 95% PI (Figs 3 and 5).

**Fig 5:**
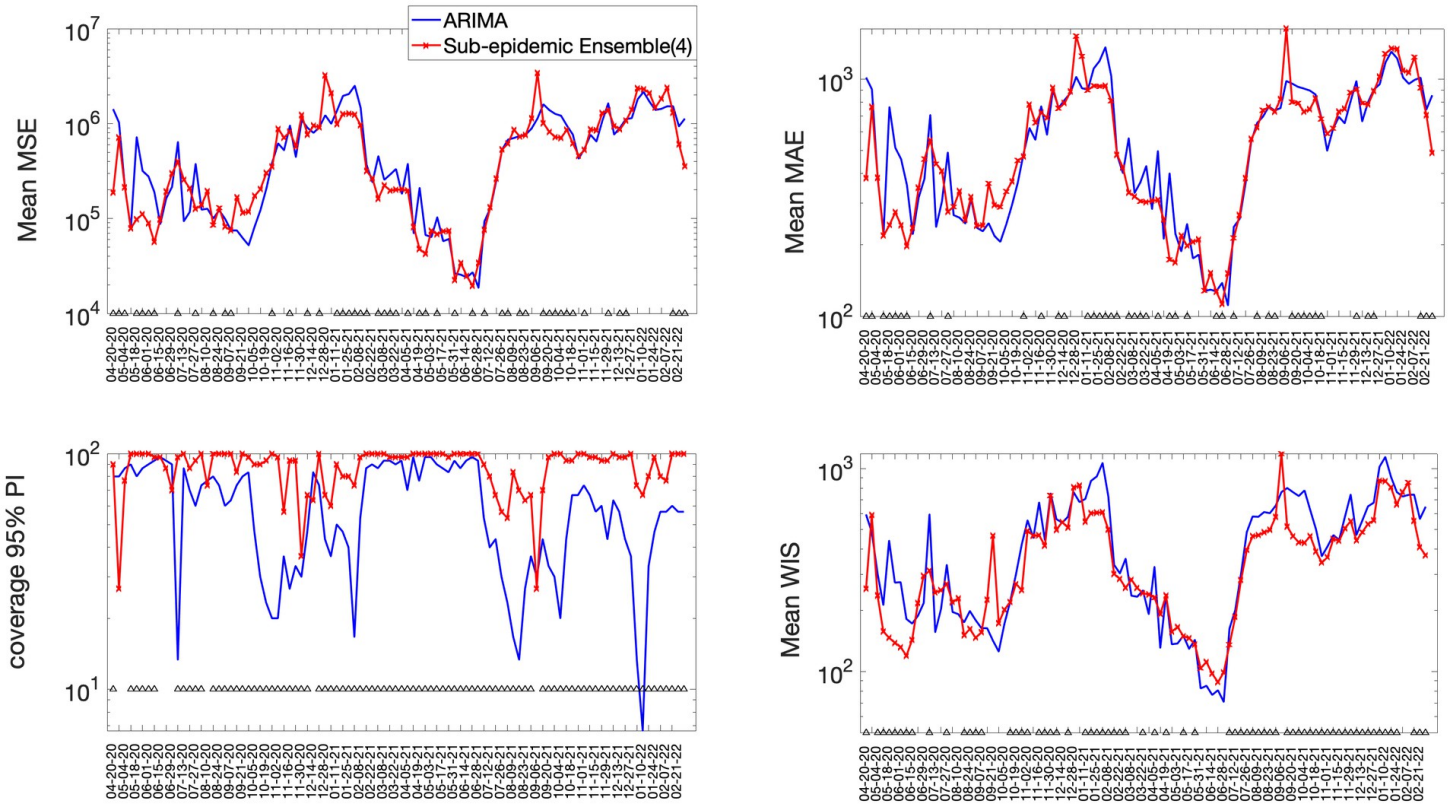
Forecasting performance metrics for the ARIMA and Ensemble(4) models across 98 30-day forecasts. The symbol (^) indicates weekly forecasts where the Ensemble(4) model outperforms the ARIMA model. For instance, the Ensemble(4) outperformed the ARIMA model 69.4% of the time in terms of the WIS and 91.8.8% of the time in terms of the coverage rate of the 95% PI (Figs 3 and 5).

The best fit sub-epidemic model and three ensemble models constructed using the top-ranking sub-epidemic models (Ensemble(2), Ensemble(3), Ensemble(4)) consistently outperformed the ARIMA models in terms of the weighted interval score (WIS) and the coverage of the 95% prediction interval across the 10, 20 and 30-day short-term forecasts (Table 2). For instance, for 30-day forecasts, the average WIS ranged from 377.6 to 421.3 for the sub-epidemic models, whereas it ranged from 439.29 to 767.05 for the ARIMA models. Across 98 short-term forecasts, the Ensemble(4) outperformed the (log) ARIMA model 66.3% of the time and the ARIMA model 69.4% of the time in 30-day ahead forecasts in terms of the WIS (Figs 4 and 5). Similarly, the 95% PI coverage ranged from 82.2% to 88.2% for the sub-epidemic models, whereas it ranged from 58% to 60.3% for the ARIMA models in 30-day forecasts. In terms of the coverage of the 95% PI, the Ensemble(4) outperformed the (log) ARIMA model 89.8% of the time and the ARIMA model 91.8% of the time (Figs 4 and 5). Forecasting performance generally improved as the number of top-ranking sub-epidemic models included in the ensemble increased (Table 1). The Ensemble(4) model consistently yielded the best performance in terms of the metrics that account for the uncertainty of the predictions.

**Table 2:** Mean forecasting performance metrics for the sub-epidemic models (ensemble weights are proportional to the reciprocal of the AICc) and the ARIMA models across 98 sequential weekly calibration periods of the daily time series of COVID-19 deaths in the USA from 20-April-2020 through 22-February 2022. Values highlighted in bold correspond to the best performance metrics.

Model	Mean absolute error (MSE)	Mean squared error (MAE)	Percentage coverage of the 95% prediction interval	Weighted Interval Score (WIS)
**10 days ahead**				
Top-ranked sub-epidemic model	551740.00	535.16	87.14	352.00
Ensemble(2) model	504560.00	516.44	88.88	331.83
Ensemble(3) model	491020.00	513.39	**89.29**	328.00
Ensemble(4) model	491740.00	513.14	**89.39**	**326.56**
(log) ARIMA model	**424880.00**	**458.72**	42.45	365.19
ARIMA model	430070.00	467.18	43.06	380.47
**20 days ahead**				
Top-ranked sub-epidemic model	646880.00	570.34	85.15	382.90
Ensemble(2) model	576700.00	544.35	88.57	354.04
Ensemble(3) model	558890.00	540.71	**89.59**	350.73
Ensemble(4) model	557130.00	539.30	**89.44**	**346.83**
(log) ARIMA model	591980.00	536.22	51.07	422.41
ARIMA model	**538690.00**	**528.87**	55.05	404.92
**30 days ahead**				
Top-ranked sub-epidemic model	749560.00	613.75	82.18	421.29
Ensemble(2) model	670740.00	586.52	87.35	383.36
Ensemble(3) model	650790.00	584.20	**88.20**	382.79
Ensemble(4) model	**644270.00**	**579.77**	**88.16**	**377.64**
(log) ARIMA model	818530.00	621.58	57.99	767.05
ARIMA model	656480.00	591.93	60.34	439.29

*The Ensemble(*i*) model incorporates the top *i* ranked sub-epidemic models in the ensemble as described in the text.

In terms of the metrics based on point estimate information, the ARIMA models showed lower overall MSE or MAE compared to the sub-epidemic models in 10 and 20-day forecasts. However, the Ensemble(4) achieved the best forecasting performance in 30-day forecasts (Table 2). Overall, the forecasting performance deteriorated at longer forecasting horizons across all models considered in our study.

**Fig 6:**
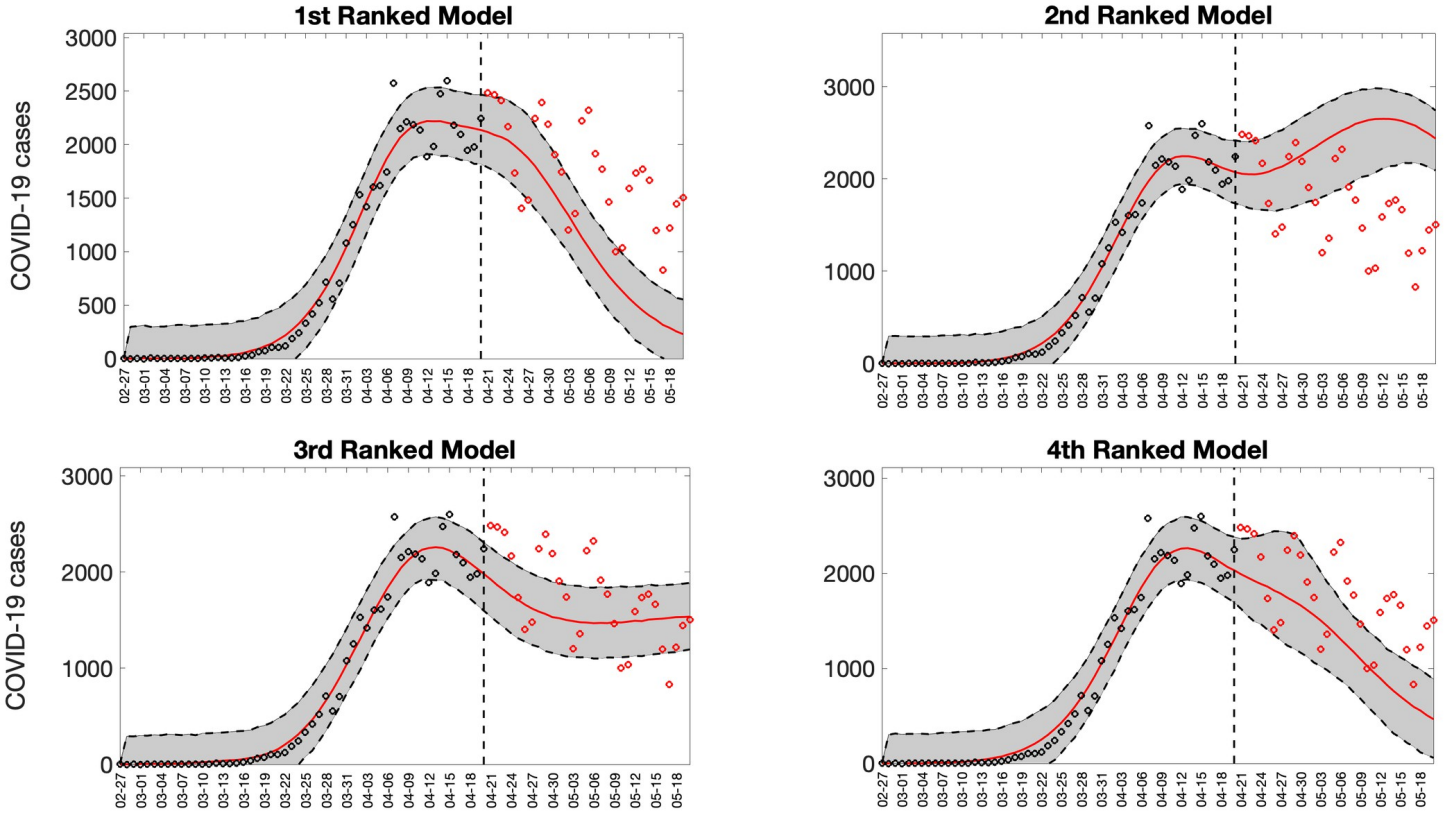
Representative 30-day forecasts of the top-ranking sub-epidemic models to the daily curve of COVID-19 deaths in the USA from 20-April-2020 to 20-May-2020. The model fit (solid line) and 95% prediction interval (shaded area) are also shown. The vertical line indicates the start time of the forecast. Circles correspond to the data points. These four top-ranking models support forecasts with diverging trajectories even though they yield similar fits to the calibration period. For instance, the 1^st^ ranked sub-epidemic model predicts a decline in the mortality curve, whereas the 2^nd^ ranked model predicts a stable pattern during the next 30 days.

**Fig 7:**
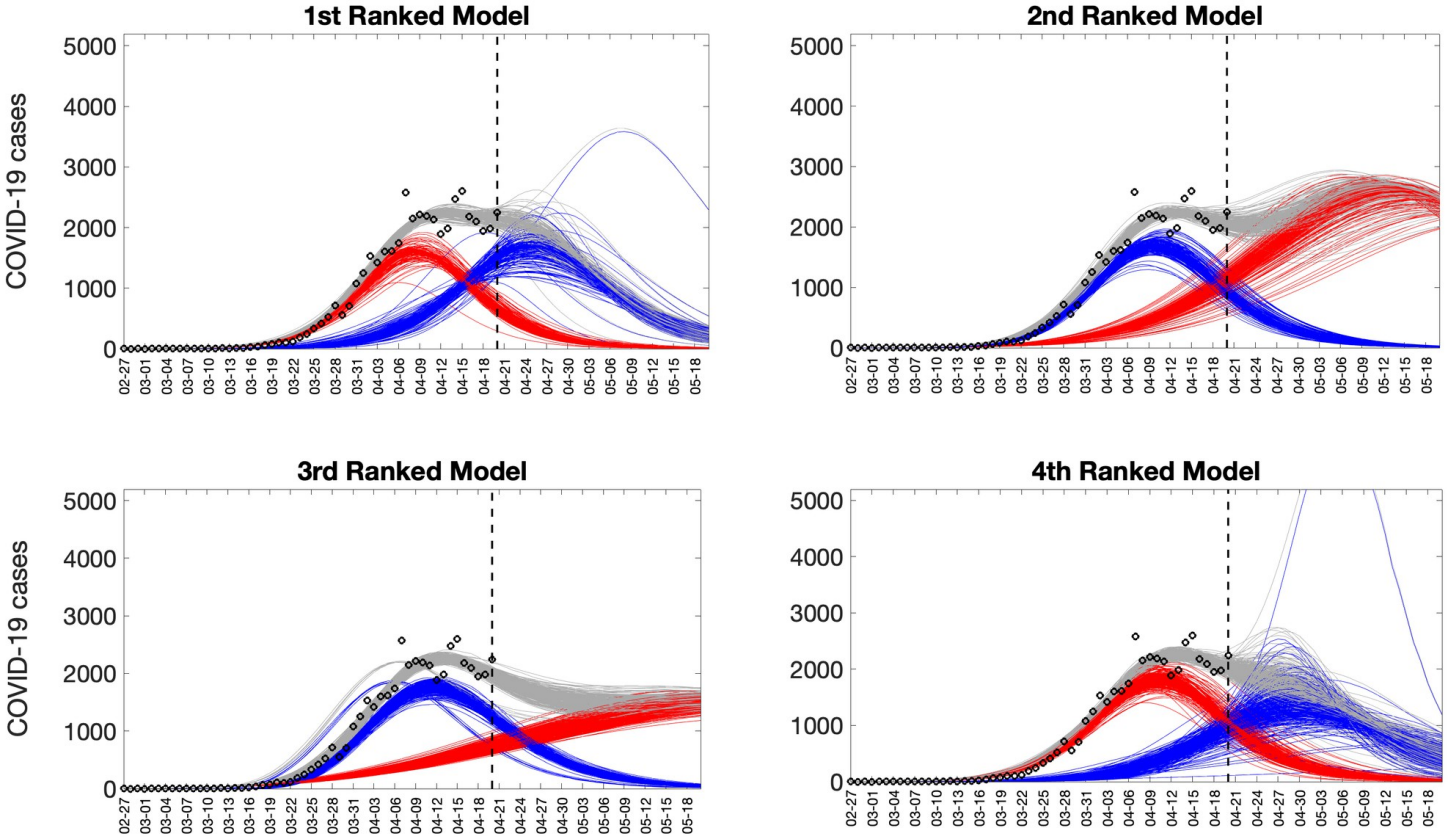
Representative sub-epidemic profiles of the forecasts derived from the top-ranking sub-epidemic models to the daily curve of COVID-19 deaths in the USA from 20-April-2020 to 20-May-2022. The model fit (solid line) and 95% prediction interval (shaded area) are also shown. Black circles correspond to the calibration data. Blue and red curves represent different sub-epidemics of the epidemic wave profile. Gray curves correspond to the overall epidemic trajectory obtained by aggregating the sub-epidemic curves. The vertical line indicates the start time of the forecast.

**Fig 8:**
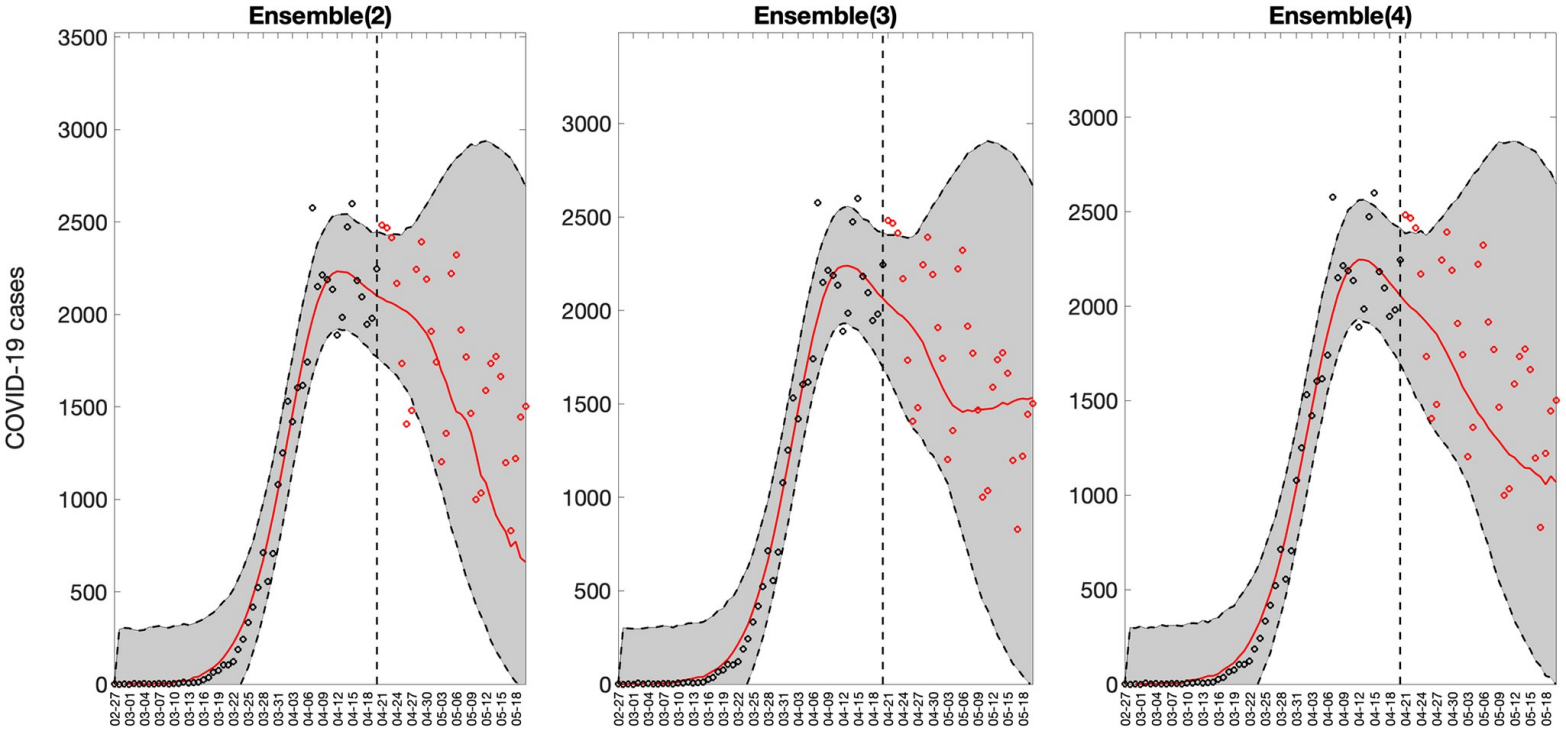
Representative sub-epidemic ensemble model forecasts (Ensemble(2), Ensemble(3), Ensemble(4)) of COVID-19 deaths in the USA from 20-April-2020 to 20-May-2022. Circles correspond to the data points. The model fits (solid line), and 95% prediction intervals (shaded area) are shown. Circles correspond to the data points. The vertical line indicates the start time of the forecast.

Representative 30-day forecasts of the top-ranking sub-epidemic models to the daily curve of COVID-19 deaths in the USA from 20-April-2020 to 20-May-2022 are shown in Fig 6. The corresponding sub-epidemic profiles of the forecasts are shown in Fig 7. These models support forecasts with diverging trajectories even though they yield similar fits to the calibration period. For instance, the top-ranked sub-epidemic model predicts a decline in the mortality curve, whereas the second-ranked model predicts a stable pattern during the next 30 days (Fig 6). The corresponding forecasts generated from three ensemble models (Ensemble(2), Ensemble(3), Ensemble(4)) built from the top-ranking sub-epidemic models are shown in Fig 8. The individual 30-day ahead predictions across 98 forecasting periods generated by the Ensemble(4) and the ARIMA models are available in the GitHub repository [30].

In sensitivity analyses, defining ensemble weights as proportional to the relative likelihood did not perform better than the ensemble models generated using weights proportional to the reciprocal of the AIC_c_. Moreover, the rank of the ensemble models was not affected by the type of weights (Table 3).

**Table 3:** Mean forecasting performance metrics for the sub-epidemic models (ensemble weights were based on the relative likelihood) and the ARIMA models across 98 sequential weekly calibration periods of the daily time series of COVID-19 deaths in the USA from 20-April-2020 through 22-February 2022. Values highlighted in bold correspond to the best performance metrics.

Model	Mean absolute error (MSE)	Mean squared error (MAE)	Percentage coverage of the 95% prediction interval	Weighted Interval Score (WIS)
**10 days ahead**				
Top-ranked sub-epidemic model	551740.00	535.16	87.14	352.00
Ensemble(2) model	548540.00	534.14	**87.25**	348.66
Ensemble(3) model	547220.00	533.51	**87.25**	**347.99**
Ensemble(4) model	546350.00	533.23	**87.35**	**347.60**
(log) ARIMA model	**424880.00**	**458.72**	42.45	365.19
ARIMA model	430070.00	467.18	43.06	380.47
**20 days ahead**				
Top-ranked sub-epidemic model	646880.00	570.34	85.15	382.90
Ensemble(2) model	640240.00	567.90	**85.71**	377.27
Ensemble(3) model	640960.00	568.45	**85.71**	**376.67**
Ensemble(4) model	639280.00	567.74	**85.56**	**376.36**
(log) ARIMA model	591980.00	536.22	51.07	422.41
ARIMA model	**538690.00**	**528.87**	55.05	404.92
**30 days ahead**				
Top-ranked sub-epidemic model	749560.00	613.75	82.18	421.29
Ensemble(2) model	744130.00	612.63	**82.65**	**414.72**
Ensemble(3) model	745230.00	613.21	**82.59**	**414.54**
Ensemble(4) model	743020.00	612.48	**82.52**	**414.16**
(log) ARIMA model	818530.00	621.58	57.99	767.05
ARIMA model	**656480.00**	**591.93**	60.34	439.29

## Discussion

Our ensemble sub-epidemic modeling approach outperformed individual top-ranking sub-epidemic models and a set of ARIMA models in weekly short-term forecasts covering the national trajectory of the COVID-19 pandemic in the USA from the early growth phase up until the Omicron-dominated wave. This framework has demonstrated reliable forecasting performance across different pandemic phases, from the early growth phase characterized by exponential or sub-exponential growth dynamics to plateaus and new disease surges driven by the relaxation of social distancing policies or the emergence of new variants. Importantly, we found that forecasting performance consistently improved for the ensemble sub-epidemic models that incorporated a higher number of top-ranking sub-epidemic models. The ensemble model incorporating the top four ranking sub-epidemic models consistently yielded the best performance, particularly in terms of the coverage rate of the 95% prediction interval and the weighted interval score.

Our findings support the power of ensemble modeling approaches (e.g.,[14–17]). Our ensemble modeling framework derived from a family of sub-epidemic models demonstrated improved performance as the number of top-ranking sub-epidemic models included in the ensemble increased. Prior studies have documented the potential of ensemble models to enhance forecasting performance during multi-epidemic periods [14]. For instance, in the context of influenza, one study utilized "weighted density ensembles" for predicting timing and severity metrics and found that the performance of the ensemble model was comparable to that of the top individual model, albeit the ensemble’s forecasts were more stable across influenza seasons [17]. In the context of dengue in Puerto Rico, another study found that forecasts derived from Bayesian averaging ensembles outperformed a set of individual models [25]. Results from the US COVID-19 Forecasting Hub CDC were consistent with our findings in that a multimodel ensemble frequently outperformed the set of individual models.

We also evaluated short-term forecasting performance by a set of ARIMA models, as prior studies have underscored the value of ARIMA models in epidemic forecasting [31], by providing a relatively simple and transparent approach to forecasting. For instance, in the context of forecasting influenza-like illness in the USA, a set of ARIMA models provided reasonably accurate short-term forecasts during the 2016/17 influenza season [32]. In another forecasting study during multiple influenza seasons in the USA, an ARIMA model yielded similar short-term forecasting performance compared to other models based on the mechanistic SIR modeling framework [33]. ARIMA models have also been used for spatial prediction of the COVID-19 epidemic [34,35]. Another study [36] showed that the ARIMA model is more effective than the Prophet time series model for forecasting COVID-19 prevalence. Finally, it is worth noting that the US COVID-19 Forecast Hub did not include an ARIMA model in its set of evaluated models [37]. Therefore, it is interesting to assess how ARIMA models perform in the context of the COVID-19 pandemic in the US.

Prior work has underscored the need to assess alternative ways of constructing ensembles from individual models [14,16]. We explored two ways of constructing the ensembles by relying on the AIC_c_ or the relative likelihood associated with the individual models. We found that the short-term forecasting performance achieved by the ensemble models was not significantly affected by the type of ensemble weights used to construct them. However, performance using ensemble weights based on the reciprocal of the AIC_c_ was slightly better. Further research could explore how different weighting strategies influence the forecasting performance of ensemble modeling approaches.

Short-term forecasting is an essential attribute of the models. As prior studies have underscored, longer-term forecasts are of value, but their dependability varies inversely with the time horizon. Our 20 and 30-day forecasts are most valuable for monitoring, managing, and informing the relaxation of social distancing requirements. The early detection of potential disease resurgence can signal the need for strict distancing controls, and the reports of cases can identify the geographic location of incubating sub-epidemics.

Our study is not exempt from limitations. Although ensemble *n*-sub-epidemic modeling is effective in forecasting the COVID-19 pandemic using the CSSE dataset [38], a single dataset is not sufficient to demonstrate the general effectiveness of any method. It will be important to compare the approach with competing methods on other datasets and infectious diseases in the future. Our analysis relied on daily time series data of COVID-19 deaths in the USA, which is inherently noisy due to heterogeneous data reporting at fine spatial scales (i.e., county-level) [39]. Noisy data complicate the ability of any mathematical model to identify meaningful signals about the impact of transmission dynamics and control interventions. To deal with the high noise levels in the data, we fitted the models to smoothed time series rather than the actual daily series, as described in the parameter estimation section. Other forecasting studies, including the US COVID-19 Forecasting Hub, have relied on weekly death counts to address this issue [37]. Beyond the COVID-19 pandemic, there is a need to establish benchmarks to systematically assess forecasting performance across a diverse catalog of mathematical models and epidemic datasets involving multiple infectious diseases, social contexts, and spatial scales.

While our analysis demonstrated the accuracy of our ensemble sub-epidemic modeling framework in forecasting the COVID-19 pandemic, the same framework could be readily used to forecast other epidemics irrespective of the type of disease and spatial scale involved. Beyond infectious diseases, this framework could also be used to forecast other biological and social growth processes, such as the epidemics of lung injury associated with e-cigarette use or vaping and the viral spread of information through social media platforms.

In summary, our ensemble sub-epidemic models provided reliable short-term forecasts of the trajectory of the COVID-19 pandemic in the USA involving multiple waves and outcompeted a set of ARIMA models. The forecasting performance of the ensemble models improved with the number of top-ranking sub-epidemic models included in the ensemble. This framework could be readily applied to investigate the spread of epidemics and pandemics beyond COVID-19 and in a range of problems in nature and society that would benefit from short-term predictions.

## Materials and methods

### Data

We used daily COVID-19 deaths reported in the USA from the publicly available data tracking system of the Johns Hopkins Center for Systems Science and Engineering (CSSE) from 27 February 2020 to 30 March 2022 [38]. The data is updated on the CSSE webpage daily at 23:59 (UTC) and read from the daily case report. The data is also publicly available in the GitHub repository [30].

### *n*-sub-epidemic model

We model epidemic trajectories comprised of one or more overlapping and asynchronous sub-epidemics. The sub-epidemics are used as building blocks to characterize more complex epidemic trajectories. The mathematical equation for the sub-epidemic building block is the 3-parameter generalized-logistic growth model (GLM), which has performed well in short-term forecasts of single outbreak trajectories for different infectious diseases, including COVID-19 [40–42]. This model is given by the differential equation:

$$\frac{dC\left(t\right)}{dt}=rC^{p}\left(t\right)\left(1-\frac{C\left(t\right)}{K_{0}}\right),$$

where $\frac{dC\left(t\right)}{dt}$ describes the curve of daily deaths over time *t*. The cumulative curve at time *t* is given by *C*(*t*), while *r* is a positive parameter denoting the growth rate per unit of time, *K*_0_ is the final outbreak size, and *p* ∈ [0, 1] is the "scaling of growth" parameter which allows the model to capture early sub-exponential and exponential growth patterns. If = 0, this equation describes a constant number of new deaths over time, while *p* = 1 indicates that the early growth phase is exponential. Intermediate values of *p* (0 < *p* < 1) describe early sub-exponential (e.g., polynomial) growth dynamics.

An *n*-sub-epidemic trajectory comprises *n* overlapping sub-epidemics and is given by the following system of coupled differential equations:

$$\frac{dC_{i}\left(t\right)}{dt}=A_{i}\left(t\right)r_{i}C_{i}{}^{p_{i}}\left(t\right)\left(1-\frac{C_{i}\left(t\right)}{K_{0}{}_{{}_{i}}}\right),$$

where *C*_*i*_(*t*) tracks the cumulative number of deaths for sub-epidemic *i*, and the parameters that characterize the shape of the *i*_*th*_ sub-epidemic are given by $\left(r_{i},p_{i},K_{0}{}_{{}_{i}}\right)$, for *i* = 1, …, *n*. Thus, the 1-sub-epidemic model is equivalent to the generalized growth model described above. When *n* > 1, we model the onset timing of the (*i* + 1)_*th*_ sub-epidemic, where (*i* + 1) ≤ *n*, by employing an indicator variable given by *A*_*i*_(*t*) such that the (*i* + 1)_*th*_ sub-epidemic is triggered when the cumulative curve of the *i*_*th*_ sub-epidemic exceeds *C*_*thr*_.

The (*i* + 1)_*th*_ sub-epidemic is only triggered when $C_{thr}\leq K_{0_{i}}$. We have:

$$A_{i}\left(t\right)=\{\begin{array}{c} 1,\;C_{i-1}\left(t\right)>C_{thr}\\ \;0,\;\text{Otherwise} \end{array}\;i=2,\ldots n,$$

where *A*_1_(*t*) = 1 for the first sub-epdemic. Hence, the total number of parameters needed to model an *n*-sub-epidemic trajectory is given by 3*n* + 1. The initial number of deaths is given by *C*_1_(0) = *I*_0_, where *I*_0_ is the initial number of deaths in the observed data. The cumulative curve of the *n*-sub-epidemic trajectory is given by:

$$C_{tot}\left(t\right)=\sum_{i=1}^{n}C_{i}\left(t\right).$$

The *n*-sub-epidemic wave model can characterize diverse epidemic patterns, including epidemic plateaus where the epidemic stabilizes at a high level for an extended period, epidemic resurgences where the number of cases increases again after a low incidence period, and epidemic waves characterized by multiple peaks.

### Parameter estimation

To reduce the noise in the original data due to artificial reasons such as the weekend effects, we use the 7-day moving average of daily death series to fit the *n*-sub-epidemic model. Let

$$y_{t_{j}}=y_{t_{1},}y_{t_{2}},\ldots ,y_{t_{n_{d}}}\;\text{where}\;j=1,2,\ldots ,n_{d}$$

denote the smoothed daily COVID-19 death series of the epidemic trajectory based on the moving average. Here, *t*_*j*_ are the time points for the time series data, *n*_*d*_ is the number of observations, and each $y_{t_{j}}$, j = 1,2,…, *n*_*d*_, is the average of the death counts at the neighboring seven days (*t*_*j*−3_, *t*_*j*−2_, *t*_*j*−1_, *t*_*j*_, *t*_*j*+1_, *t*_*j*+2_, *t*_*j*+3_). We will use this smoothed data to estimate a total of 3*n* + 1 model parameters, namely $\Theta =\left(C_{thr},r_{1},p_{1},K_{0}{}_{{}_{1}},\ldots ,r_{n},p_{n},K_{0}{}_{{}_{n}}\right)$. Let *f*(*t*, Θ) denote the expected curve of new COVID-19 deaths of the epidemic’s trajectory. We can estimate model parameters by fitting the model solution to the observed data via nonlinear least squares [43] or via maximum likelihood estimation assuming a specific error structure [44]. For nonlinear least squares, this is achieved by searching for the set of parameters $\hat{\Theta }$ that minimizes the sum of squared differences between the observed data $y_{t_{j}}=y_{t_{1},}y_{t_{2}}\ldots ‥y_{t_{n_{d}}}$ and the model mean, corresponding to *f*(*t*, Θ). That is,$\Theta =\left(C_{thr},r_{1},p_{1},K_{0}{}_{{}_{1}},\ldots ,r_{n},p_{n},K_{0}{}_{{}_{n}}\right)$ is estimated by $\hat{\Theta }=\text{arg}\;\text{min}\;\sum_{j=1}^{n_{d}}{\left(f\left(t_{j},\Theta \right)-y_{t_{j}}\right)}^{2}$. We estimate parameter *C*_*thr*_ through simple discretization of its range of plausible values. Our estimation procedure consists of two steps. First, for each *C*_*thr*_, we search for the set of parameters $\left(r_{1},p_{1},K_{0}{}_{{}_{1}},\ldots ,r_{n},p_{n},K_{0}{}_{{}_{n}}\right)$ to minimize the sum of squared errors (SSE). Then we choose the *C*_*thr*_ and the corresponding estimates of other parameters leading to the minimum SSE as the best fit.

This parameter estimation method weights each data point equally and does not require a specific distributional assumption for *y*_*t*_, except for the first moment *E*[*y*_*t*_] = *f*(*t*_*i*_; *Θ*). That is, the mean of the observed data at time *t* is equivalent to the expected count (e.g., number of deaths) denoted by *f*(*t*, *Θ*) at time *t* [45]. This method yields asymptotically unbiased point estimates regardless of any misspecification of the variance-covariance error structure. Hence, the estimated model mean $f\left(t_{i},\hat{\Theta }\right)$ yields the best fit to observed data $y_{t_{i}}$ in terms of squared L2 norm. We can use the *fmincon* function in MATLAB to set the optimization problem.

To quantify parameter uncertainty, we follow a parametric bootstrapping approach which allows the computation of standard errors and related statistics in the absence of closed-form formulas [46]. We generate *B* bootstrap samples from the best-fit model $f\left(t,\hat{\Theta }\right)$, with an assumed error structure, to quantify the uncertainty of the parameter estimates and construct confidence intervals. Typically, the error structure in the data is modelled using a probability model such as the Poisson or negative binomial distribution. Because the time-series data we are fitting to involve large counts, the Poisson or negative binomial distribution can be well approximated by a normal distribution for large numbers. So, using the best-fit model $f\left(t,\hat{\Theta }\right)$, we generate *B*-times replicated simulated datasets of size *n*_*d*_, where the observation at time *t*_*j*_ is sampled from a normal distribution with mean $f\left(t,\hat{\Theta }\right)$ and variance $\frac{\sum_{j=1}^{n_{d}}{\left(f\left(t_{j},\hat{\Theta }\right)-y_{t_{j}}\right)}^{2}}{n_{d}-\left(3n+1\right)}$. Next, we refit the model to each of the *B* simulated datasets to re-estimate parameters for each. The new parameter estimates for each realization are denoted by ${\hat{\Theta }}_{b}$ where *b* = 1,2,…,*B*. Using the sets of re-estimated parameters $\left({\hat{\Theta }}_{b}\right)$, it is possible to characterize the empirical distribution of each estimate, calculate the variance, and construct confidence intervals for each parameter. The resulting uncertainty around the model fit can similarly be obtained from $\left(t,{\hat{\Theta }}_{1}\right)$, $f(t,{\hat{\Theta }}_{2}),\ldots ,f(t,{\hat{\Theta }}_{B})$.

### Model-based forecasts with quantified uncertainty

Forecasting the model, $f(t,\hat{\Theta })$, *h* days ahead provides an estimate for $f(t+h,\hat{\Theta })$. The uncertainty of the forecasted value can be obtained using the previously described parametric bootstrap method. Let

$$f\left(t+h,{\hat{\Theta }}_{1}\right),\;f\left(t+h,{\hat{\Theta }}_{2}\right),\ldots ,f\left(t+h,{\hat{\Theta }}_{B}\right)$$

denote the forecasted value of the current state of the system propagated by a horizon of *h* time units, where ${\hat{\Theta }}_{b}$ denotes the estimation of parameter set Θ from the *b*_*th*_ bootstrap sample. We can use these values to calculate the bootstrap variance as the measure of the uncertainty of the forecasts and use the 2.5% and 97.5% percentiles to construct the 95% prediction intervals (PI).

### Model selection

To select the top-ranked sub-epidemic models, we analyze the *AIC*_*c*_ values of the set of best fit models that include the 1-subepidemic model as well as the 2-subepidemic models with different values of *C*_*thr*_. We ranked the models from best to worst according to their *AIC*_*c*_ values, which is given by [47, 48]:

$$AIC_{c}=n_{d}log\left(SSE\right)+2m+\frac{2m\left(m+1\right)}{n_{d}-m-1}$$

where $SSE=\sum_{j=1}^{n_{d}}{\left(f\left(t_{j},\hat{\Theta }\right)-y_{t_{j}}\right)}^{2}$, *m* = 3*n* + 1 is the number of model parameters, and *n*_*d*_ is the number of data points. The *AIC*_*c*_ for the parameter estimation from the nonlinear least-squares fit implicitly assumes normal distribution for error.

We selected the top four ranking sub-epidemic models for further analysis. We used them to construct three ensemble sub-epidemic models, which we refer to as Ensemble(2), Ensemble(3), and Ensemble(4). The following section describes the process of constructing these ensemble models from the top-ranking sub-epidemic models.

### Constructing Ensemble Models from top-ranking models

**Fig 9:**
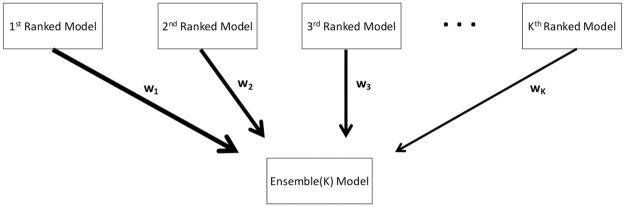
Schematic diagram of the construction of the ensemble model from the weighted combination of the highest-ranking sub-epidemic models as deemed by the $AIC_{c_{i}}$ for the *i*-th model where $AIC_{c_{1}}\leq \cdots \leq AIC_{c_{I}}$ and *i* = 1, …, *I*. An ensemble derived from the top-ranking *I* models is denoted by Ensemble(I).

Ensemble models that combine the strength of multiple models may exhibit significantly enhanced predictive performance (e.g., [14–17]). We generate ensemble models from the weighted combination of the highest-ranking sub-epidemic models as deemed by the $AIC_{c_{i}}$ for the *i*-th ranked model where $AIC_{c_{1}}\leq \ldots \leq AIC_{c_{I}}$ and *i* = 1, …, *I*. An ensemble derived from the top-ranking *I* models is denoted by Ensemble(*I*) and illustrated in Fig 9. Thus, Ensemble(2) and Ensemble(3) refer to the ensemble models generated from the weighted combination of the top-ranking 2 and 3 models, respectively. We compute the weight *w*_*i*_ for the *i*-th model, *i* = 1, …, *I*, where ∑ *w*_*i*_ = 1 as follows:

$$w_{i}=\frac{\frac{1}{AIC_{c_{i}}}}{\frac{1}{AIC_{c_{1}}}+\frac{1}{AIC_{c_{2}}}+\ldots +\frac{1}{AIC_{c_{I}}}}\;\text{for}\;\text{all}\;i=1,2,\ldots ,I,$$

and hence *w*_*I*_ ≤ ⋯ ≤ *w*_1_.

The estimated mean curve of daily COVID-19 deaths for the Ensemble(*I*) model is:

$$f_{ens\left(I\right)}\left(t\right)=\overset{I}{\underset{i=1}{\sum }}w_{i}f_{i}\left(t,{\hat{\Theta }}^{\left(i\right)}\right)$$

where given the training data, ${\hat{\Theta }}^{\left(i\right)}$ denotes the set of estimated parameters, and $f_{i}\left(t,{\hat{\Theta }}^{\left(i\right)}\right)$ denotes the estimated mean curve of daily COVID-19 deaths, for the *i*-th model. Accordingly, we compute the weighted average and sample the bootstrap realizations of the forecasts for each model to construct the 95% CI or PI using the 2.5% and 97.5% quantiles, as previously described to derive ensembles of different growth models in ref. [16]. Our MATLAB (The Mathworks, Inc) code for model fitting and forecasting is publicly available in the GitHub repository.

As a sensitivity analysis, we also investigated how the ensemble sub-epidemic models performed when the ensemble weights were proportional to the relative likelihood (*l*) rather than the reciprocal of the AIC_c_. Let *AIC*_*min*_ denote the minimum *AIC* from the set of models. The relative likelihood of model *i* is given by $l_{i}=e^{\left((AIC_{min}-AIC_{i}\right)/2)}$ [49]. We compute the weight *w*_*i*_ for the *i*-th model where ∑ *w*_*i*_ = 1 as follows:

$$w_{i}=\frac{l_{i}}{l_{1}+l_{2}+\ldots +l_{I}}\;\text{for}\;\text{all}\;i=1,2,\ldots ,I,$$

and hence *w*_*I*_ ≤ ⋯≤ w_1_.

### Auto-regressive integrated moving average models (ARIMA)

We also generated short-term predictions of the pandemic trajectory using ARIMA models to compare their performance with the sub-epidemic models. ARIMA models have frequently been employed to forecast financial trends [50–52] and weather [53–55]. The ARIMA (p, d, q) process is given by

$$\phi \left(B\right){\left(1-B\right)}^{d}y_{t}=c+\theta \left(B\right)\epsilon_{t}$$

or equivalently as *ϕ*(*B*) (1 − *B*)^*d*^ (*y*_*t*_ − μ*t*^*d*^/*d*!) = θ(*B*)ϵ_*t*_, where p is the order of the AR model, d is the degree of differencing, q is the order of the MA model, {*ϵ*_*t*_} is a white noise process with mean 0 and variance σ^2^, and B denotes the backshift operator. The p-order polynomial ϕ(*z*) = 1 − ϕ_1_*z* − ⋯ − ϕ_*p*_*z*^*p*^ and the q-order polynomial d θ(*z*) = 1 − θ_1_ *z* − ⋯ −*θ*_1_*z*^*q*^ are assumed to have no roots inside the unit circle to ensure causality and invertibility. The constant *c* = *μ*(1 − *ϕ*_1_ − ⋯ − *ϕ*_*p*_), and μ is the mean of (1 − *B*)^*d*^*y*_*t*_. When d = 0, *μ* is the mean of *y*_*t*_.

The *auto*.*arima* function in the R package "forecast" is used to select orders and build the model [56]. First, the degree of differencing 0 ≤ *d* ≤ 2 is selected based on successive KPSS unit-root tests [57], which test the data for a unit root; if the test result is significant, the differenced data is tested for a unit root; and this procedure is repeated until the first insignificant result is obtained. Then given d, the orders p and q are selected based on the *AIC*_*c*_ for the d-times differenced data. For d = 0 or d = 1, a constant will be included if it improves the *AIC*_*c*_ value; for d>1, the constant *μ* is fixed at 0 to avoid the model having a quadratic or higher order trend, which is dangerous when forecasting. The final model is fitted using the maximum likelihood estimation.

To guarantee that the forecasted values and prediction intervals are above zero, we use the following two strategies. In the first one, we conduct the ARIMA order selection and model fitting using the log-transformed data. Then we take the exponential of the forecasted values and the PI bounds to predict the incident death counts and get the PIs. We refer to this approach as the (log) ARIMA throughout the manuscript. In the second case, the negative values are set as zero. Then, it is possible that the actual coverage probability of such PIs can be smaller than the nominal value (95%). We refer to this approach as ARIMA throughout the manuscript.

### Forecasting strategy and performance metrics

We conducted short-term forecasts using the top-ranking *n*-sub-epidemic model (1 ≤ *n* ≤ 2) and three ensemble models constructed with the top-ranking sub-epidemic models, namely Ensemble(2), Ensemble(3), and Ensemble(4). For comparison, we also generated short-term forecasts using the previously described ARIMA models. Overall, we conducted 588 forecasts across models.

Using a 90-day calibration period for each model, we conducted 98 weekly sequential 10-day, 20-day and 30-day forecasts from 20 April 2020 to 28 February 2022, spanning five pandemic waves. This range of forecasting horizons is comparable to that investigated in prior COVID-19 forecasting studies [37]. This period covers the latter part of the early spring wave, a summer wave in 2020, a fall-winter 2020/2021 wave, the summer-fall wave in 2021, and the winter 2022 wave.

To assess the forecasting performance, we used four performance metrics: the mean absolute error (MAE), the mean squared error (MSE), the coverage of the 95% prediction intervals, and the weighted interval score (MIS) [58]. The *mean absolute error* (MAE) is given by:

$$\text{MAE}=\frac{1}{N}\overset{N}{\underset{h=1}{\sum }}\left|f\left(t_{h},\hat{\Theta }\right)-{\tilde{y}}_{t_{h}}\right|.$$

Here ${\tilde{y}}_{t_{h}}$ is the time series of the original death counts (unsmoothed) of the *h*-time units ahead forecasts, where *t*_*h*_ are the times for the sample data [59]. Similarly, the *mean squared error* (MSE) is given by:

$$\text{MSE}=\frac{1}{N}\overset{N}{\underset{h=1}{\sum }}{\left(f\left(t_{h},\hat{\Theta }\right)-{\tilde{y}}_{t_{h}}\right)}^{2}.$$

We also employed two metrics that account for prediction uncertainty: the *coverage rate of the 95% PI* e.g., the proportion of the observations that fall within the 95% PI as well as the *weighted interval score* (WIS) [58, 60] which is a proper score. The WIS and the coverage rate of the 95% PIs take into account the uncertainty of the predictions, whereas the MAE and MSE only assess the closeness of the mean trajectory of the epidemic to the observations [61].

Recent epidemic forecasting studies have embraced the Interval Score (IS) for quantifying model forecasting performance [18, 24, 37, 62]. The WIS provides quantiles of predictive forecast distribution by combining a set of ISs for probabilistic forecasts. An IS is a simple proper score that requires only a central (1−α)×100% PI [58] and is described as

$$IS_{\alpha }\left(F,y\right)=\left(u-l\right)+\frac{2}{\alpha }\times \left(l-y\right)\times 1\left(y<l\right)+\frac{2}{\alpha }\times \left(y-u\right)\times 1\left(y>u\right).$$

In this equation **1** refers to the indicator function, meaning that **1**(*y* < *l*) = 1 if *y* < *l* and

0 otherwise. The terms *l* and *u* represent the $\frac{\alpha }{2}$ and $1-\frac{\alpha }{2}$ quantiles of the forecast *F*. The IS consists of three distinct quantities:

1. The sharpness of *F*, given by the width *u* − *l* of the central (1 − *α*) × 100% PI.
2. A penalty term $\frac{2}{\alpha }\times \left(l-y\right)\times 1\left(y<l\right)$ for the observations that fall below the lower end point *l* of the (1 − *α*) × 100% PI. This penalty term is directly proportional to the distance between *y* and the lower end *l* of the PI. The strength of the penalty depends on the level *α*.
3. An analogous penalty term $\frac{2}{\alpha }\times \left(y-u\right)\times 1\left(y>u\right)$ for the observations falling above the upper limit *u* of the PI.

To provide more detailed and accurate information on the entire predictive distribution, we report several central PIs at different levels (1 − *α*_1_) < (1 − *α*_2_) < ⋯ < (1 − *α*_*K*_) along with the predictive median, *m*, which can be seen as a central prediction interval at level 1 − *α*_0_ ⟶ 0. This is referred to as the WIS, and it can be evaluated as follows:

$$WIS_{\alpha_{0:K}}\left(F,y\right)=\frac{1}{K+\frac{1}{2}}.\left(w_{0}.\left|y-m\right|+\sum_{k=1}^{K}w_{k}.IS_{\alpha_{k}}\left(F,y\right)\right)$$

where, $w_{k}=\frac{\alpha_{k}}{2}$ for *k* = 1,2, ….*K* and $w_{0}=\frac{1}{2}$. Hence, WIS can be interpreted as a measure of how close the entire distribution is to the observation in units on the scale of the observed data [10,37].
